# Effects of IgM Allotype Suppression on Serum IgM Levels, B-1 and B-2 Cells, and Antibody Responses ir Allotype Heterozygous F1 Mice

**DOI:** 10.1155/1994/45728

**Published:** 1994

**Authors:** Ann Marie Hamilton, John F. Kearney

**Affiliations:** 1 Division of Developmental and Clinical Immunology Department of Microbiology University of Alabama Birmingham Alabama 35294 USA; 2 378 Wallace Tumor Institute University of Alabama Alabama 35294 USA

**Keywords:** B lymphocytes, IgM, allotype, IgD, CD5, B220, phosphorylcholine, dextran

## Abstract

IgM allotype heterozygous F1 mice were independently suppressed for Igh6a or Igh6b to evaluate the contribution of B-1 and B-2 cells to natural serum IgM levels and Ab responses. B-2 B cells expressing IgM of the suppressed allotype were evident in the spleens of suppressed mice 4 to 6 weeks after cessation of the suppression regimen, whereas B-1 B cells of the suppressed allotype were undetectable for up to 9 months. Although serum IgM of the suppressed allotype was initially depleted in mice suppressed for either allotype, by 7 months of age, there were detectable levels of IgM of the suppressed allotype in the serum; however, the levels were significantly below that found in nonsuppressed mice. When mice were immunized with either the T-independent or T-dependent form of phosphorylcholine, those suppressed for either allotype, and consequently depleted of B-1 B cells of that allotype, did not respond with phosphorylcholine-specific IgM of the suppressed allotype. In contrast, when mice were immunized with α1-3 dextran, the Igh6a allotype-suppressed mice were able to produce dextran-specific IgM of that allotype. These results show that allotype-bearing B-1 cells of both allotypes can be effectively suppressed by this suppression protocol and this produces long-lasting effects on B-1 cell levels and serum IgM of the suppressed allotype. These observations reflect the derivation of the majority of B-1 cells from fetal-neonatal precursors, which cannot be replaced by newly emerging B-2 cells of adult origin. Their ablation by antibody treatment results in permanent alterations to the adult B-cell repertoire.


*Developmental Immunology*, 1994, Vol 4, pp. 27–41
Reprints available directly from the publisher
Photocopying permitted by license only

© 1994 Harwood Academic Publishers GmbH
Printed in Singapore

### Effects of IgM Allotype Suppression on Serum IgM Levels, B-1 and B-2 Cells, and Antibody Responses ir Allotype Heterozygous F1 Mice

ANN MARIE HAMILTON[†] and JOHN F. KEARNEY[*†]

[†]*Division of Developmental and Clinical Immunology, Department of Microbiology, University of Alabama, Birmingham, Alabama 35294*

IgM allotype heterozygous F1 mice were independently suppressed for Igh6a or Igh6b to evaluate the contribution of B-1 and B-2 cells to natural serum IgM levels and Ab responses. B-2 B cells expressing IgM of the suppressed allotype were evident in the spleens of suppressed mice 4 to 6 weeks after cessation of the suppression regimen, whereas B-1 B cells of the suppressed allotype were undetectable for up to 9 months. Although serum IgM of the suppressed allotype was initially depleted in mice suppressed for either allotype, by 7 months of age, there were detectable levels of IgM of the suppressed allotype in the serum; however, the levels were significantly below that found in nonsuppressed mice. When mice were immunized with either the T-independent or T-dependent form of phosphorylcholine, those suppressed for either allotype, and consequently depleted of B-1 B cells of that allotype, did not respond with phosphorylcholine-specific IgM of the suppressed allotype. In contrast, when mice were immunized with $\alpha$1–3 dextran, the Igh6a allotype-suppressed mice were able to produce dextran-specific IgM of that allotype. These results show that allotype-bearing B-1 cells of both allotypes can be effectively suppressed by this suppression protocol and this produces long-lasting effects on B-1 cell levels and serum IgM of the suppressed allotype. These observations reflect the derivation of the majority of B-1 cells from fetal-neonatal precursors, which cannot be replaced by newly emerging B-2 cells of adult origin. Their ablation by antibody treatment results in permanent alterations to the adult B-cell repertoire.

KEYWORDS: B lymphocytes, IgM, allotype, IgD, CD5, B220, phosphorylcholine, dextran.

### INTRODUCTION

The primary and secondary Ab responses to a variety of defined Ag have been well characterized in mice with respect to Id profiles, responder versus nonresponder strains, and the B-cell subsets involved in the Ab response. The primary response to the T-independent Ag phosphorylcholine (PC) is characterized by the dominant expression of the TEPC15 (T15) Id (Cosenza and Köhler, 1972; Sher and Cohn). Although most strains of mice are able to make Ab specific for PC following immunization, there is evidence that the ability to respond and the magnitude of this response is regulated by the heavy-chain locus ($IgC_H$) (Lieberman et al., 1974; Sigal et al., 1977; Cancro et al., 1978; Stall et al., 1986). Other Ab responses have also been linked to the $IgC_H$ locus and are expressed only in mice bearing particular $IgC_H$ allotypes. For example, the anti-$\alpha$1-3 dextran (DEX) response characterized by the J558 Id is absent in C57BL/6 mice and other strains bearing the $IgC_H^b$ allotype (Blomberg et al., 1972). Whereas these responses have been described in genetically uniform strains of mice, the interplay between B-1 and B-2 B cell subsets and the relationship between B-1 and B-2 cells as precursors or as regulatory cells in these $IgC_H$-linked Ab responses in F1 hybrid mice have not been studied.

B-1 B cells are defined as B cells expressing high levels of surface (s) IgM, low levels of sIgD, Mac-1$^+$, and IL5R$^+$; in adult mice, they are primarily located in the peritoneal cavity rather than in spleen and lymph nodes. B-1 B cells are further subdivided into

*Corresponding author. Present address: 378 Wallace Tumor Institute, University of Alabama, Alabama 35294.

B-1a, those which express the CD5 marker, and B-1b, which do not. The majority of natural serum IgM is derived from B-1 B cells (Hardy, 1992). B-2 B cells are $IgM^{lo}$, $IgD^{hi}$, and $Fc\varepsilon R^{+}$; they constitute the majority of B cells in the secondary lymphoid organs—spleen, lymph nodes, and Peyer's patches (Hardy, 1992).

In this study, we have determined whether Ab responses to the antigens PC and DEX reside solely within the B-1 or B-2 B cell populations, and whether B-1 B cells influence the idiotypic profile of the responses. The experimental model uses $IgC_H$ allotype heterozygous mice that have been treated neonatally with either of the Igh6 allotype-specific monoclonal antibodies (mAb). Previous studies have shown that B cell production in mice can be ablated by chronic administration of anti-μ Ab (Cooper et al., 1980). Lalor and colleagues (1989b) demonstrated that neonatal suppression of the paternal IgM allotype depleted B-1a B cells of the suppressed allotype for up to 10 months after birth. However, the B-2 and B-1b B cell populations expressing the paternal IgM allotype returned to normal levels in the adult, similar to previous studies with total anti-μ suppression. We have extended the use of this model by independently suppressing the maternal or paternal IgM allotype in F1 mice, providing an *in vivo* model with the potential to investigate the relative contributions of B-1 versus B-2 B cells of each parental allotype to specific Ab responses and to serum Ig levels in mice expressing two genetically distinct Ig allotypes.

### RESULTS

### Clearance of RS-3.1 and MB86 Occurs at Different Rates

F1 mice derived from Igh6a dams and Igh6b sires were subjected to IgM allotype suppression and, to determine the length of time the suppressing Ab could be detected, their sera were analzyed at various intervals after birth for the presence of either RS-3.1 or MB86, the allotype-specific mAb used in the suppression protocol. There were no detectable levels of RS-3.1 in the serum of the mice treated with this Ab 4 weeks previously (Table 1). However, the mice that received MB86 had 3–20 μg/ml of this Igh6b-specific mAb in their circulation at 4 and 5 weeks after the final injection. We have duplicated these experiments with the reverse pairs of parent, Igh6a dams, and Igh6b sires, and obtained essentially the same patterns of suppression (data not shown).

### Differential Recovery of B-1 and B-2 Cells following Allotype-Specific Suppression

CB6 F1 mice were analyzed at various ages for the presence of B-1 and B-2 B cells of both allotypes in the spleen and peritoneal cavity. The purpose of this analysis was to determine the time at which B-2 and B-1 B cells reappeared in the suppressed animals following clearance of allotype-specific mAb (see earlier).

At 16 days after birth, which corresponded to 2 days after the final anti-allotype injection, all splenic B cells of the suppressed allotype were completely depleted, as shown by the total absence of $B220^{+}$ cells expressing the suppressed allotype (Fig. 1). The total spleen cell number was approximately equal between the nonsuppressed ($78 \times 10^6$) and Igh6a- ($60 \times 10^6$) or Igh6b- ($80 \times 10^6$) suppressed CB6 F1 mice. However, the frequency of $B220^{lo}\mu^{-}$ pre-B cells in the Igh6a- and Igh6b-suppressed mice was increased by 31–54% compared to nonsuppressed littermates (data not shown). At 6 weeks after birth, approximately 20% of splenic B cells from Igh6a-suppressed CB6 F1 mice were found to express Igh6a as compared to 33% in the nonsuppressed

TABLE 1
Clearance of the Allotype-Specific mAb in Allotype-Suppressed Mice[a]

| | Serum Levels of Allotype Specific mAb (μg/ml)[b] | | | |
|---|---|---|---|---|
| Suppressed for | 4 weeks | 6 weeks | 7 weeks | 14 weeks |
| Igh6a[c] | 12.7 ± 7.7 | 0 ± 0 | 0 ± 0 | 0 ± 0 |
| Igh6b[d] | 25.7 ± 14.0 | 11.9 ± 7.8 | 2.8 ± 2.8 | 0 ± 0 |

[a]Allotype-suppressed mice were bled at various times after cessation of the suppression protocol and sera tested in an ELISA for the presence of the allotype-specific mAb. In all of these experiments, the dams were Igh6a and sires Igh6b for the CB6 (F1) offspring.
[b]Values represent the mean ±S.D. for 4 to 14-week-old mice.
[c]Sera was titrated on Igh6a-coated plates. After overnight incubation, alkaline phosphatase-labeled goat anti-mouse $IgG_1$ was used to capture any previously bound RS-3.1 mAb. Color was developed using p-nitrophenyl phosphate as a substrate, and the levels of RS-3.1 were measured using ELISA LITE.
[d]Sera were titrated on Igh6b-coated plates and analyzed for the presence of MB86 as described earlier.

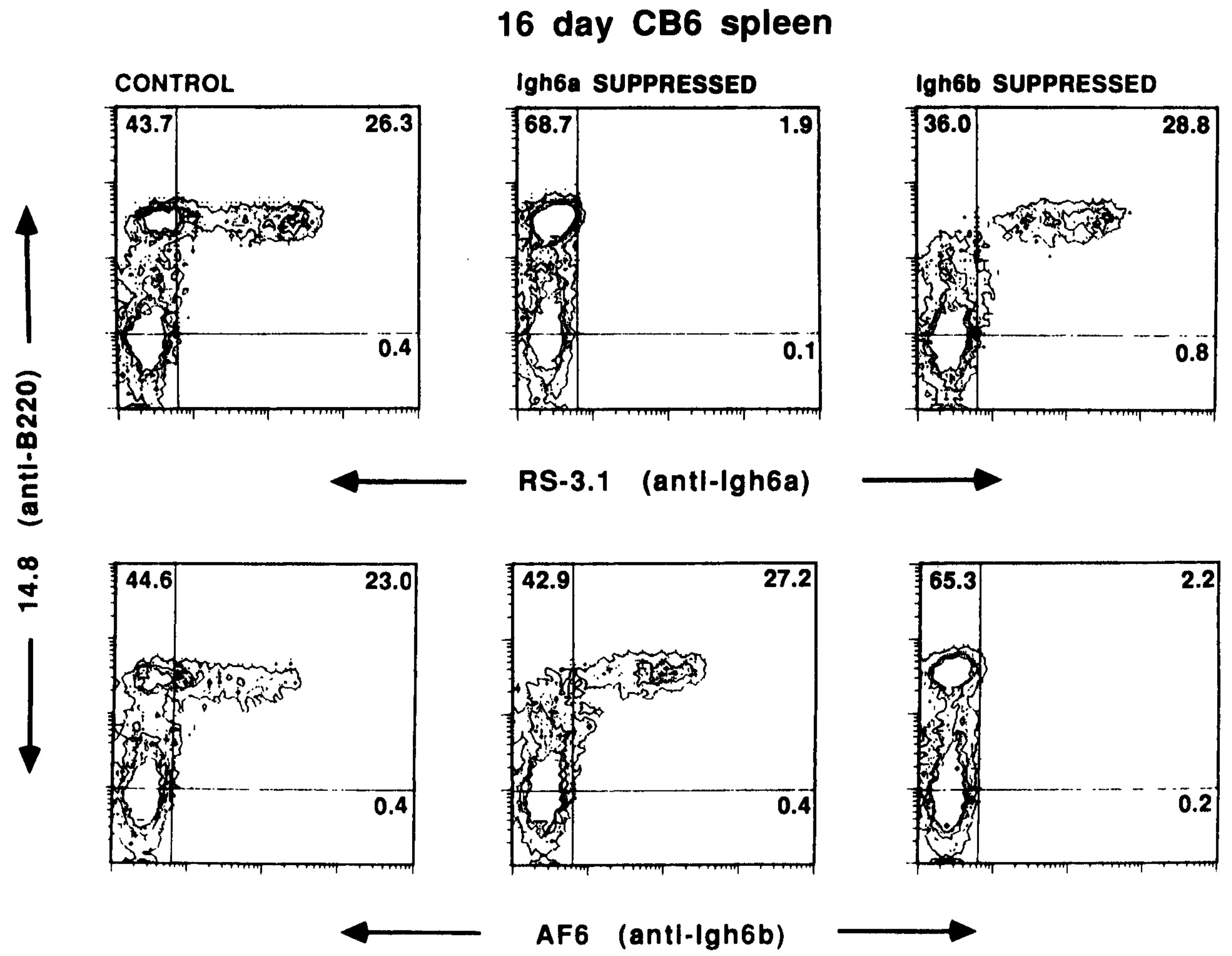

FIGURE 1. Effectiveness of allotype suppression assessed 2 days after the final injection of allotype-specific mAb. Splenocytes from control (left panels), Igh6a-suppressed (center panels), and Igh6b-suppressed (right panels) mice were stained with FITC-labeled anti-Igh6a (upper panels) and anti-Igh6b (lower panels), and biotin-labeled anti-B220 followed by SA-PE and analyzed by two-color flow cytometry using a FACScan to confirm the absence of B cells expressing the suppressed allotype. The percentage of positive cells is indicated.

group (Fig. 2A). However, the Igh6b-suppressed CB6 F1 mice still did not possess any B-2 B cells expressing the suppressed allotype in the spleen at this time. B-2 cells expressing the Igh6b allotype were not found in the spleen of Igh6b-suppressed CB6 F1 mice until 8 weeks after birth (data not shown).

Evaluation of the B lymphocyte composition of the peritoneal cavity with a panel of mAb previously shown to delineate B-1 and B-2 B cells (Hardy, 1992) at 6 weeks after birth revealed that there were no B-1 and very few, if any, B-2 B cells expressing IgM of the suppressed allotype in either group of IgM-suppressed mice (Fig. 2B). By 9 months after birth, B-2 B cells of the suppressed allotype were evident in the peritoneal cavity; however, there were virtually no B-1 B cells of the suppressed allotype (Fig. 3).

Immunofluorescence microscopy analysis demonstrated the lack of plasma cells expressing Igh6a or Igh6b in the spleens of CB6 F1 mice suppressed for Igh6a or Igh6b, respectively, at both 6 and 8 weeks (data not shown), which correlated with the lack of B-1 B cells of the appropriate allotype.

Data from these analyses indicated that B-2 B cells expressing the suppressed allotype reemerged between 6 to 8 weeks after birth. In contrast, B-1 B cells of either suppressed allotype were absent for up to 9 months following cessation of the allotype-specific suppressing Ab. Despite the presence of

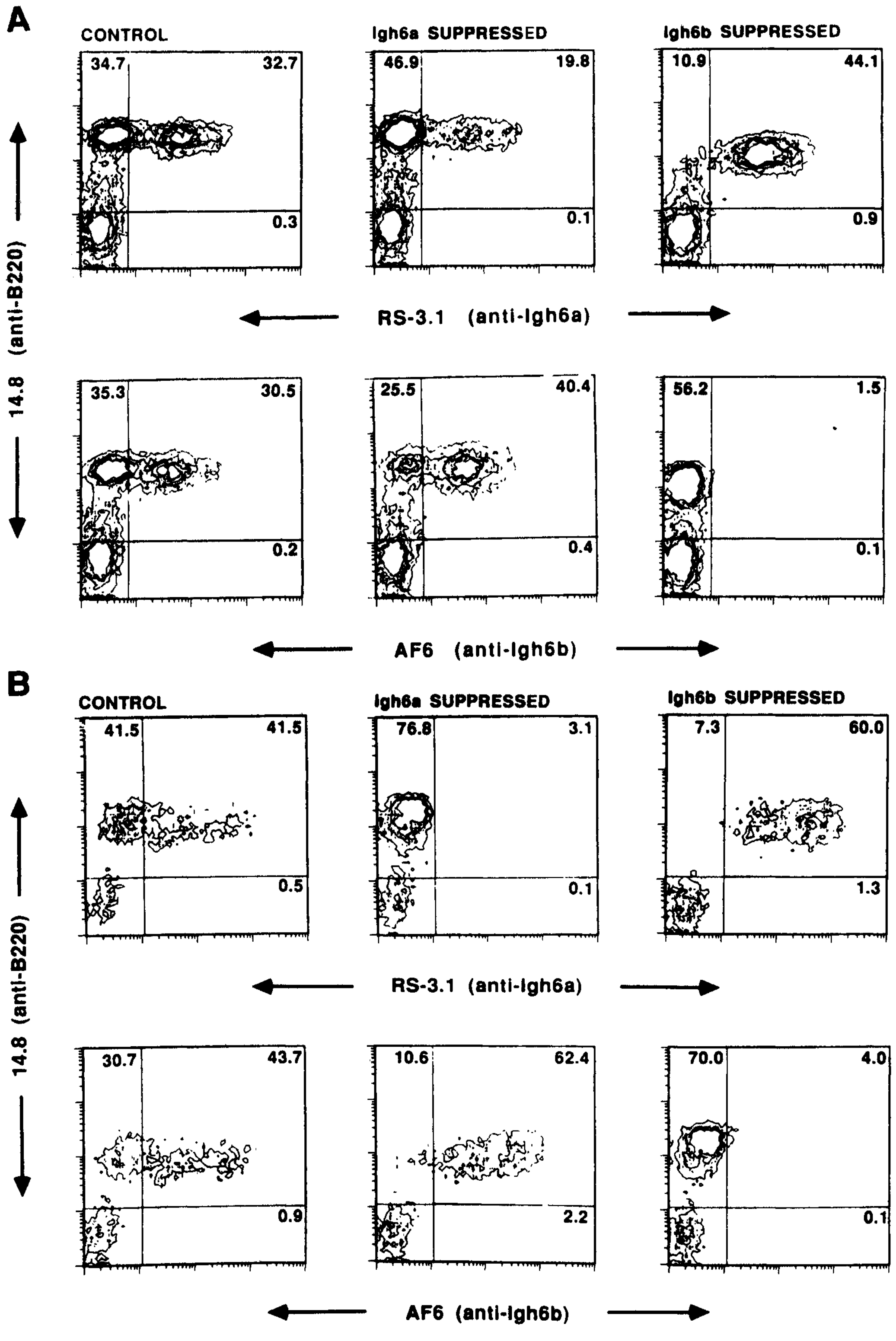

FIGURE 2. Splenic Igh6a$^+$ B-2 B cells, but not splenic Igh6b$^+$ B-2 or peritoneal Igh6a$^+$ and Igh6b$^+$ B-1 B cells, reemerge 4 weeks after cessation of the suppression regimen. (A) Splenocytes and (B) peritoneal cells from control (left panels), Igh6a-suppressed (center panels), and Igh6b-suppressed (right panels) mice were stained with FITC-labeled anti-Igh6a (upper panels of both A and B) and anti-Igh6b (lower panels of both A and B), and biotin-labeled anti-B220 followed by SA-PE to assess the reemergence of B cells expressing the suppressed allotype. The percentage of positive cells is indicated.

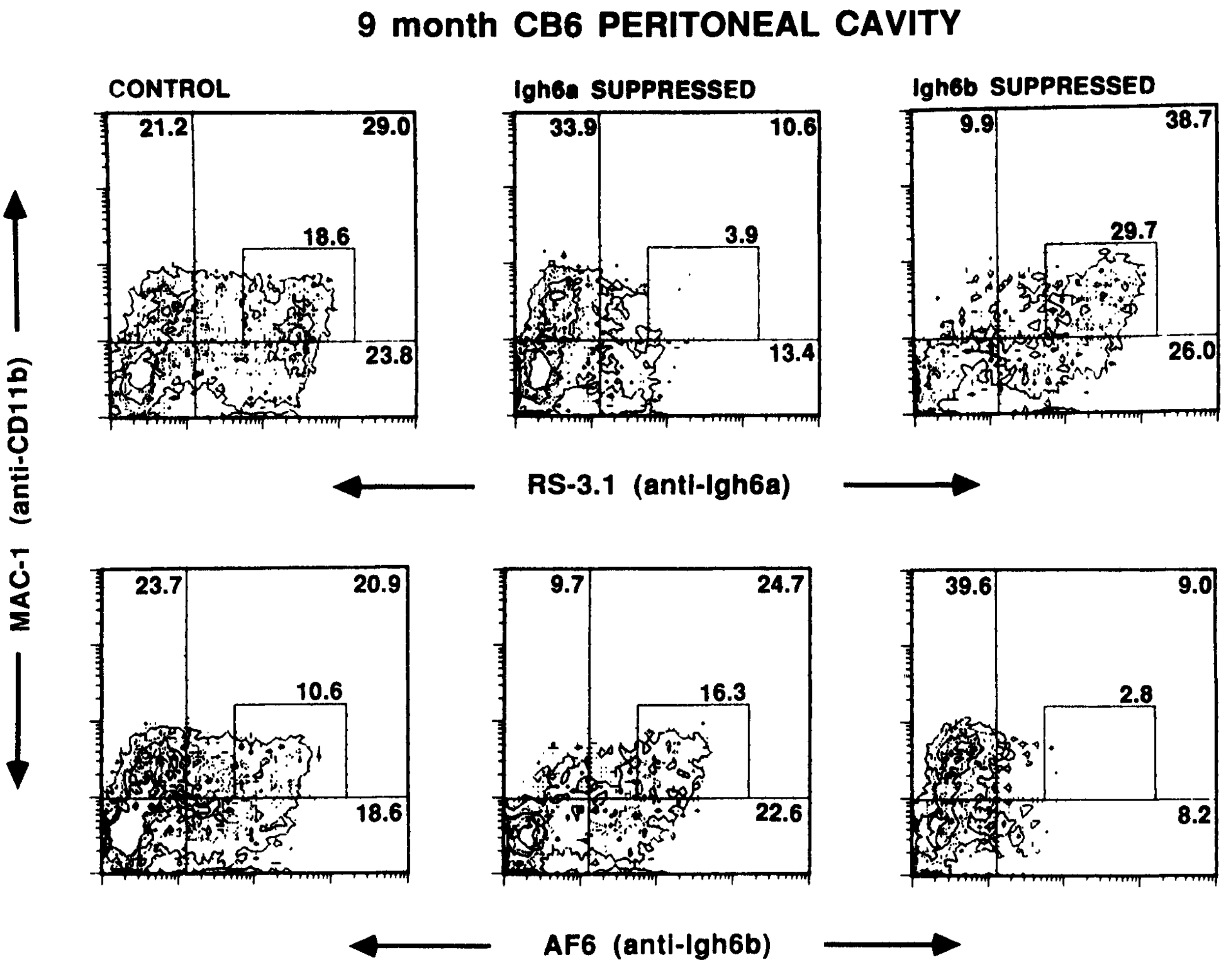

FIGURE 3. B-1 B cells expressing the suppressed allotype remain undetectable at 9 months after completion of the suppression protocol. Peritoneal lymphocytes from control (left panels), Igh6a-suppressed (center panels), and Igh6b-suppressed (right panels) mice were stained with FITC-labeled anti-Igh6a (upper panels) and anti-Igh6b (lower panels), and biotin-labeled anti-Mac-1 followed by SA-PE to determine whether B-1 B cells expressing the suppressed allotype were evident. The percentage of positive cells is indicated.

splenic B-2 B cells expressing surface IgM of the suppressed allotype, there were no plasma cells expressing cytoplasmic IgM of the suppressed allotype.

### Suppression of Igh6 B Cells Correlates with Suppression of Serum IgM of the Corresponding Igh6 Allotype

To determine whether the majority of serum IgM was derived from B-1 B cells, as suggested by previous studies (Hardy, 1992), sera from F1 mice were analyzed for the presence of IgM of both allotypes at 6 weeks, 14 weeks, and 7 months after birth.

At 6 weeks, the levels of Igh6b were approximately twice that of Igh6a in control animals (Fig. 4A). In mice suppressed for the Igh6a allotype, there was no detectable Igh6a, whereas the Igh6b levels were elevated about three fold. Conversely, in Igh6b-suppressed mice, there was no detectable IgM expressing the Igh6b allotype, whereas the Igh6a levels were increased 5.5-fold. At 14 weeks, B-2 B cells expressing either Igh6a or Igh6b were present in the control as well as both groups of suppressed mice (data not shown). At this time, the levels of IgM expressing the suppressed allotype were approximately 50 μg/ml in sera from either group of suppressed mice (Fig. 4B). At 7 months of age, mice that had been suppressed for either IgM allotype demonstrated significant levels of the suppressed allotype although at considerably lower levels than control mice, which had almost equal (400 μg/ml) amounts of both allotypes (Fig. 4C).

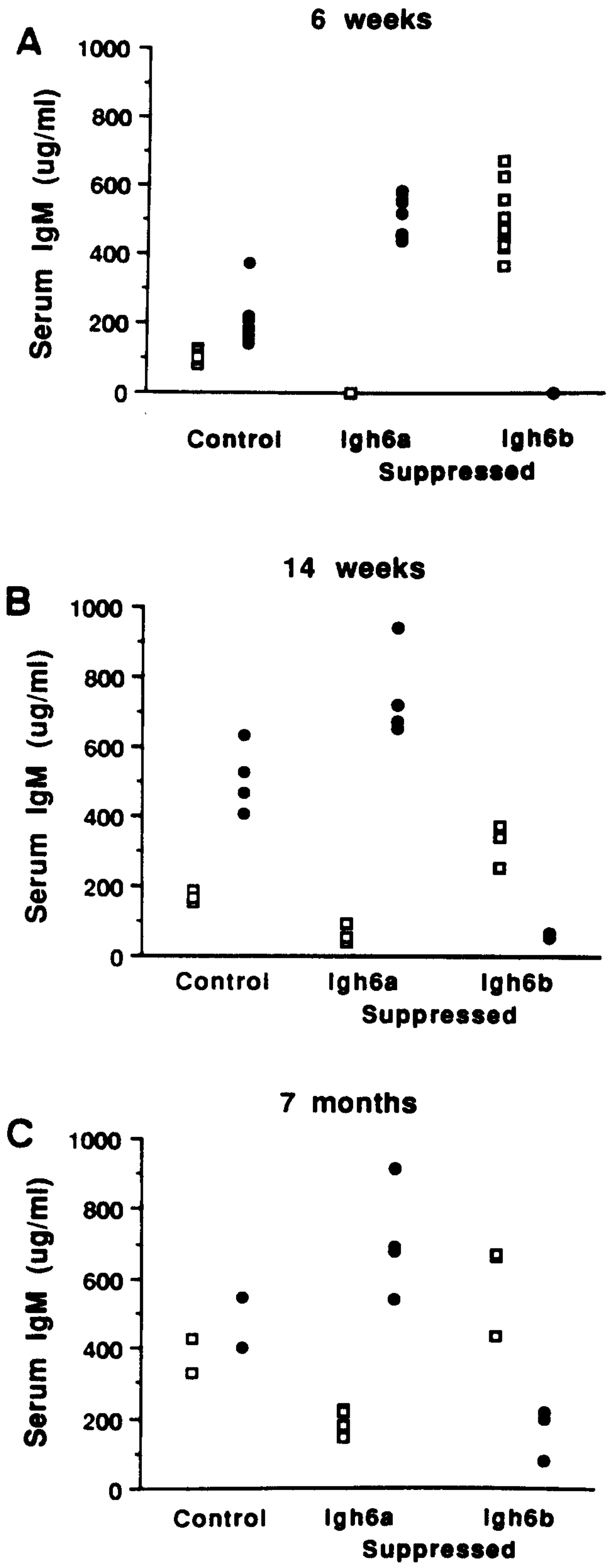

FIGURE 4. Natural serum IgM levels of the suppressed allotype reach approximately 50% of levels in nonsuppressed mice after 7 months. Sera from (A) 6-week-old, (B) 14-week-old, (C) 7-month old mice were titrated on RS-3.1- and MB86-coated plates and analyzed by an ELISA for Igh6a (open squares) and Igh6b (closed circles) levels, respectively. Values are given for individual mice in each group shown.

These findings suggest that B-2 B cells can produce small amounts (approximately 150 μg/ml) of serum IgM, compared to control mice of the same age, at extended periods after cessation of the suppression protocol. However, the majority of naturally occurring serum IgM still appears to originate from B-1 B cells, particularly in younger animals.

### B-2 B Cells Are Unable to Respond to the T-Independent Form of PC

To evaluate the ability of B-2 B cells to respond to PC, control F1 mice as well as those suppressed for either parental allotype were immunized with a heat-killed preparation of R36A. Seven days later, sera were analzyed by a quantitative ELISA for the presence of PC-specific IgM Ab of either allotype. In the control group, the level of total PC-specific IgM was approximately 25 μg/ml, 25–30% of which expressed the T15 Id, as defined by reactivity with AB1-2(Fig. 5A). Further analysis revealed that the majority of PC-specific IgM was of the Igh6b allotype indicated by the low levels of PC-specific Igh6a (Fig. 5B). Mice suppressed for the Igh6a allotype had total PC-specific IgM levels comparable to control mice, although the $T15^+$ portion of the response was slightly higher (Fig. 5A). As there were no demonstrable levels of PC-specific Igh6a Ab (Fig. 5B), the entire response was of the Igh6b allotype. Interestingly, mice suppressed for the Igh6b allotype responded to PC with IgM levels similar to the other two groups, but the T15 Id was almost completely absent (Figs. 5A and 5B). The PC-specific response in these mice was of the Igh6a allotype.

Due to the lack of AB1-2 reactivity in this latter group, the $T15^+$ portion of the PC-specific IgM response was further analyzed for expression of the $V_HS107$ heavy and $V_k22$ light chains required for the T15 Id, using a quantitative ELISA with the TC139-2 anti-T15 Id mAb. This mAb detects all PC-specific Ab that are $T15^+$ despite the presence of junctional diversity between either $V_H$-D or D-$J_H$ joins (Kearney et al., 1983). The results of this analysis indicated that Igh6b-suppressed mice did

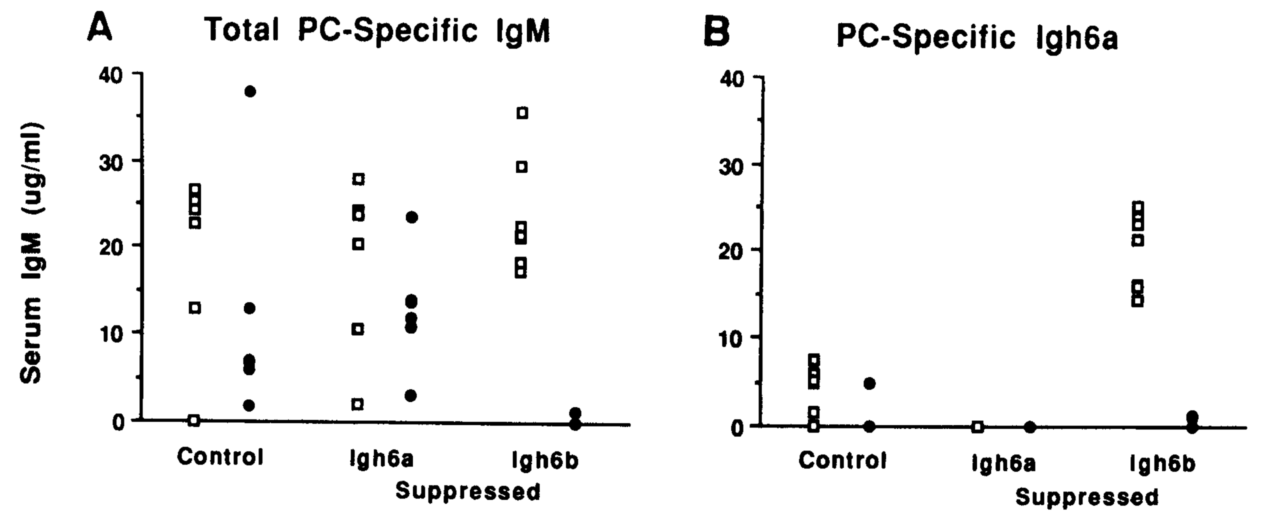

FIGURE 5. Allotype-suppressed mice only produce PC-specific Ab of the nonsuppressed allotype when challenged with the T-independent form of PC. Mice were immunized with *S. pneumoniae* (strain R36A) and sera analyzed by an ELISA for (A) total PC-specific IgM and (B) PC-specific Igh6a. Sera were titrated on plates coated with PC-BSA or AB1-2 (anti-T15 Id mAb). A polyclonal goat anti-mouse IgM Ab or RS-3.1 was used to determine the total IgM or Igh6a PC-specific Ab response, respectively. Values for PC-specific (open squares) and T15$^+$ (closed circles) Ab responses from individual control, Igh6a-, and Igh6b-suppressed mice are shown.

not produce PC-specific IgM of the correct heavy- and light-chain pair in order to generate the dominant T15 Id (Fig. 6).

At the time when these mice were immunized, B-2 B cells expressing Igh6a were evident in the spleens of Igh6a-suppressed mice (Fig. 2A). However, there were no B-1 B cells of the suppressed allotype in the peritoneal cavity of either group of suppressed mice (Fig. 2B). These data indicate that although the B-2 B cells expressing Igh6a were present in Igh6a-suppressed mice, these mice did not respond to the T-independent form of PC. Therefore, in this situation, the B-2 B cells regenerating in the suppressed mice did not respond to this antigen.

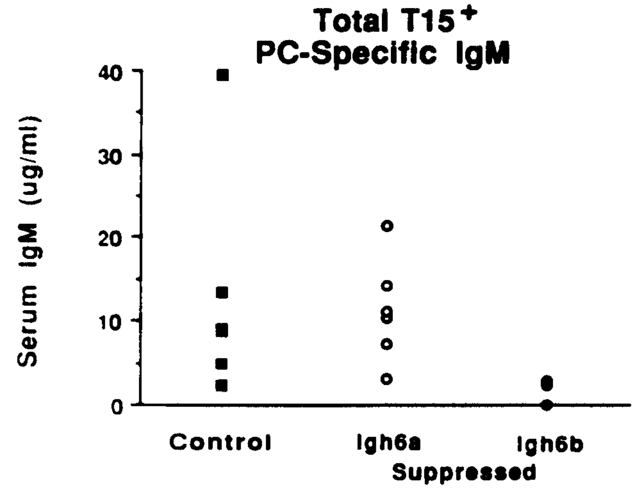

FIGURE 6. The T15$^+$ PC-specific Ab response is absent in Igh6b-suppressed mice. Sera from R36A immunized mice were analyzed for PC-specific Ab reactive with TC139-2 in an ELISA. Values shown correspond to individual control (closed squares), Igh6a- (open cirlces), and Igh6b- (closed circles) suppressed mice.

### B-2 B Cells in Allotype-Suppressed F1 Mice can Respond to $\alpha$1-3 DEX

In order to determine whether B-2 B cells can respond to DEX, F1 mice were immunized with $\alpha$1-3 DEX, and 7 days later, the serum was analyzed for the presence of DEX-specific IgM of either allotype. The total DEX-specific IgM response in nonsuppressed mice was approximately 75 µg/ml, of which 50% expressed the normally dominant J558 Id detected by reactivity with EB3-7 (Fig. 7). Mice suppressed for Igh6a demonstrated similar levels of DEX-specific and J558$^+$ IgM Ab to those in the nonsuppressed mice. Whereas the DEX-specific IgM response in Igh6b-suppressed mice was slightly elevated over the nonsuppressed mice, the percentage of J558$^+$ DEX-specific Ab was greatly reduced.

To determine the Id of the anti-DEX response in Igh6b-suppressed mice, the serum was then analyzed for the presence of the minor M104E Id. With one exception, almost 100% of the total DEX-

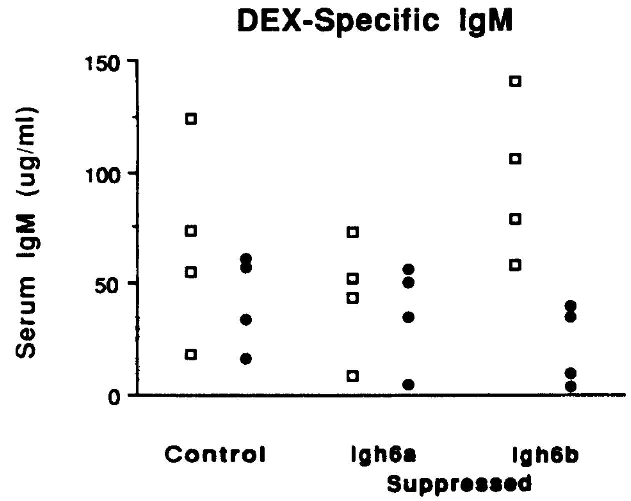

FIGURE 7. Igh6a$^+$ B-2 B cells can respond to DEX. Mice were immunized with DEX and sera analzyed by an ELISA for the presence of DEX-specific (open squares) and J558$^+$ (closed circles) IgM. Plates coated with EB3-7 (anti-J558 Id mAb) were used to determine the levels of J558 Id. Values correspond to individual control, Igh6a-, and Igh6b-suppressed mice.

specific IgM response in Igh6b-suppressed mice expressed the M104E Id (Fig. 8). The percentages of DEX-specific Ab expressing the M104E Id in nonsuppressed and Igh6a-suppressed mice were approximately 50% and 30%, respectively.

The primary IgM response to DEX in all three groups of mice was of the Igh6a allotype, as there

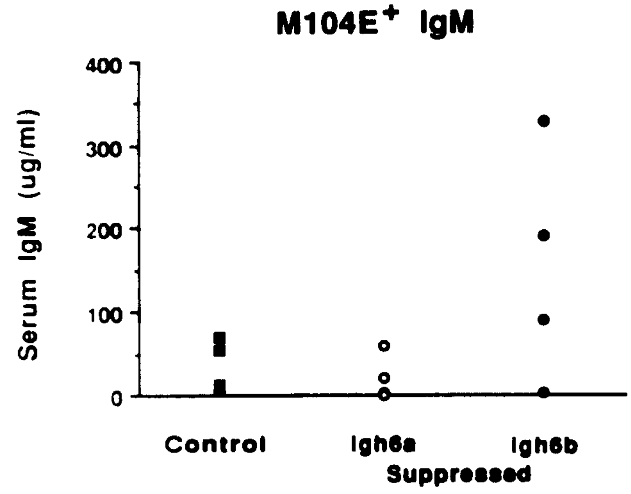

FIGURE 8. Igh6b-suppressed mice produce significant levels of M104E$^+$ DEX-specific IgM. Sera from DEX immunized mice were also analyzed by an ELISA for the presence of the minor M104E Id on plates coated with SJL18.1 (anti-M104E Id mAb). Values correspond to individual control (closed squares), Igh6a- (open circles), and Igh6b (closed circles) suppressed mice.

were no detectable levels of DEX-specific Igh6b and the amount of total $\lambda_1$ DEX-specific Ab equaled the total Igh6a DEX-specific Ab in all groups tested (data not shown). In these experiments, mice were immunized after B-2 B cells expressing Igh6a had reemerged in those animals suppressed for Igh6a, but before B-1 B cells could be found in the peritoneal cavity, so it is clear from this data that the B-2 B-cell population is able to respond to DEX.

### The Response to a T-Dependent Form of PC also Appears to Reside within the B-1 B Cell Population

F1 mice were primed with KLH and 14 days later immunized with PC-KLH, a T-dependent form of PC, to determine whether allotype suppression of B-1 B cells affects the Ab response and the idiotypic profile of the response to this Ag. Seven days after immunization, sera were analyzed for the presence of PC-specific IgM of either allotype similar to the method used for detecting PC-specific IgM in R36A immunized mice (see earlier). The total level of PC-specific IgM in nonsuppressed mice was approximately 100 μg/ml, of which almost 100% expressed the dominant T15 Id (Fig. 9A). The majority of the response was of the Igh6b allotype, as indicated by the low levels of PC-specific Igh6a, that did not express T15 (Fig. 9B). Mice suppressed for the Igh6a allotype had total PC-specific IgM levels similar to nonsuppressed mice (Fig. 9A). All of the PC-specific IgM response in these mice was of the Igh6b allotype because there was no demonstrable levels of Igh6a PC-specific IgM (Fig. 9B). Mice suppressed for Igh6b had approximately 50% lower levels of PC-specific IgM (Fig. 9A), which was entirely of the Igh6a allotype (Figs. 9A and 9B). Interestingly, the PC-specific IgM response to PC-KLH in these mice was not of the dominant T15 Id.

In $IgC_H^{a/b}$ F1 mice, the response to PC appears to be dominated by the Igh6b allotype, as illustrated in this data and the responses to the T-independent form of PC (see earlier). Therefore, suppression of the Igh6a allotype has little effect on the PC-specific Igh6b response. However, when mice were suppressed for Igh6b, they did not produce significant levels of PC-specific Igh6a and there was considerable inhibition of the dominant T15 Id. These results also indicated that the response to the T-dependent form of PC resides within the B-1 B cell population, as evidenced by the total absence of PC-specific Ab expressing the suppressed allotype.

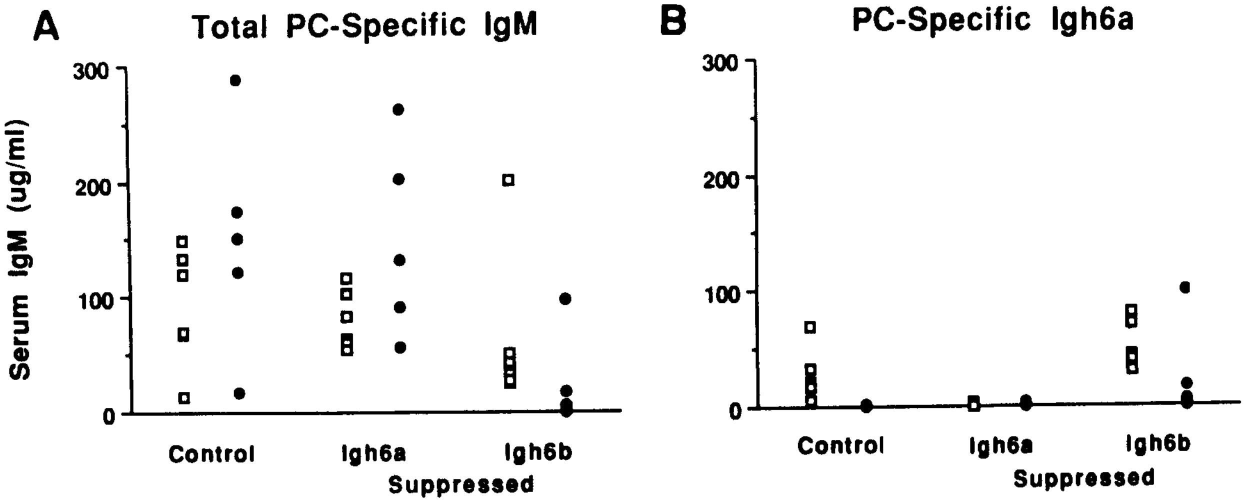

FIGURE 9. Allotype-suppressed mice only produce PC-specific Ab of the nonsuppressed allotype when challenged with the T-dependent form of PC. Mice were immunized with PC-KLH and sera analyzed by ELISA for the presence of (A) total PC-specific and (B) PC-specific Igh6a. Values for PC-specific (open squares) and T15$^+$ (closed cirlces) Ab responses from individual control, Igh6a-, and Igh6b- suppressed mice are shown.

### DISCUSSION

In order to study the interplay of B cells expressing genetically distinct Ig alleles, we chose to independently suppress either the maternal or paternal IgM allotype in F1 mice, thereby providing a means to investigate the relative contribution of B-1 and B-2 B cells of each parental allotype to specific Ab responses and to serum IgM levels. Similar to the findings of Lalor and colleagues (1989b), our results demonstrate temporary suppression of B-2 B cells expressing the suppressed allotype. In Igh6a-suppressed mice, B-2 B cells expressing Igh6a reappeared in the spleen 4 weeks after cessation of the suppression protocol. However, B-2 B cells expressing Igh6b were not found in the spleen of Igh6b-suppressed mice until 6 weeks after completion of the suppression regimen. The temporal differences in the reappearance of B-2 cells expressing either Igh6a or Igh6b in appropriately suppressed mice may be due in part to the ability of the mice to clear the allotype-specific suppressing mAb from the circulation. RS-3.1 was effectively cleared almost 3 weeks sooner then MB86, which correlates with the earlier reappearance in the spleens of these mice of B-2 B cells expressing Igh6a. We have no explanation for these differences because we propagated and purified these antibodies, both of the IgG1 isotype in the same way.

These results support previous studies that have demonstrated selective suppression of the paternal B-1 and B-2 cells in allotype heterozygous mice (Lalor et al., 1989b). When CB6 F1 mice were injected neonatally with AF6-78.25, an Igh6b-specific mAb, B-2 B cells expressing Igh6b began to reappear in the peripheral lymphoid organs shortly after the disappearance of the suppressing Ab, reaching normal numbers by 6 months. Although the authors suggest that there is complete suppression of B-1 B cells expressing Igh6b for up to 1 year, more recently (Herzenberg and Kantor, in preparation) it was shown that B-1b B cells expressing Igh6b reappeared in the peritoneal cavity at 8 months (Lalor et al., 1989a).

In contrast to these observations, we demonstrate the complete suppression of B-1 B cells for up to 9 months after cessation of the suppression protocol. Neither B-1b nor B-1a B cells reappear in either Igh6a- or Igh6b-suppressed mice, as evidenced by the complete absence of Mac-1$^+$ IgM$^{hi}$ B-1 B cells expressing the suppressed allotype in the peritoneal cavity. These data support the existence of a feedback mechanism operating in the peritoneal cavity, which regulates the development of B-1 B cells of both allotypes from progenitor cells (Lalor et al., 1989a).

A recent study in rabbits demonstrated that almost all peripheral B cells express the CD5 membrane glycoprotein suggesting that all rabbit peripheral B cells are of the B-1 type (Raman and Knight, 1992). Previous allotype suppression studies performed in Ig heterozygous rabbits (Mage, 1967;

Harrison and Mage, 1973; Eskinazi et al., 1979) produced results similar to those in mice, as reported by Lalor (1989b) and herein. In the rabbit studies, neonatal or *in utero* injection of either light-chain or heavy-chain allotype-specific Ab resulted in long-term suppression of B cells expressing that allotype. These findings further support the concept of a feedback mechanism operating to inhibit the subsequent production of B-1 B cells from progenitor cells or selection of B-1 cells into the B cell pool.

Data from several studies indicate that the majority of naturally occurring serum IgM is produced by B-1 B cells (Herzenberg et al., 1986; Förster and Rajewsky, 1987; Hardy, 1992). Our allotype-suppression model enabled us to evaluate the amounts of seurm IgM contributed by B-1 and B-2 B cells. Results in allotype-suppressed mice confirmed that the majority of serum IgM originates from B-1 B cells and that B-2 B cells produce only relatively small amounts of serum IgM after the elapse of an extended period of time from the cessation of allotype suppression. Although B-2 B cells expressing the suppressed allotype were evident in the spleen of suppressed mice at 4 to 6 weeks after completion of the suppression regimen, low levels of serum IgM of the suppressed allotype did not appear for another 6 to 8 weeks. The reason for the delay in production of serum IgM by B-2 B cells in allotype-suppressed mice is not obvious. It is likely that B-1 B cells are activated to IgM secretion by internal networks, whereas B-2 cells may be selected into the repertoire by environmental Ag (Bos et al., 1989). Therefore, production of large amounts of serum IgM in unimmunized mice by B-2 cells, as demonstrated by Thomas-Vaslin and colleagues (1992), may correlate with the degree and type of bacterial and viral flora of the mice being analyzed.

Previous cell-transfer experiments have suggested that responses to two T-independent Ag, PC and DEX, reside within the B-1 B cell subset and that B-2 B cells are unable to respond to these Ag (Förster and Rajewsky, 1987; Thomas-Vaslin et al., 1992). Furthermore, anti-Il-10 treatment of mice, which resulted in the depletion of B-1 B cells, ablated the response to DEX (Ishida et al., 1992). In our experiments, F1 allotype heterozygous mice were suppressed for either the paternal or maternal IgM allotype shortly after birth and without continuous treatment, thereby ensuring minimal manipulation of the adult immune system. Using these allotype-suppressed mice, we were then able to investigate the contribution of B-2 B cells to PC- and DEX-specific Ab responses as well as to determine the effect of B-1 B cell depletion on these responses and their idiotypic profiles. The results obtained from the analysis of these specific Ab responses shed light on the role of clonal competition between B cells for expression of each allotype and provide several explanations for previously observed phenomena in both cell-transfer studies and anti-cytokine-treated mice.

$IgC_H{}^{a/b}$ F1 mice have previously been shown to be low responders to PC (Lieberman et al., 1974). In these mice, the PC-specific IgM response appears to be dominated by the Igh6b allotype, as demonstrated by the control values in Fig. 5. Although B-2 B cells expressing the suppressed allotype were present in the appropriate group of allotype-suppressed mice, there was no detectable levels of PC-specific IgM of the suppressed allotype in the serum. These results are consistent with those obtained by Masmoudi and colleagues (1990), which demonstrated that the Ab response to the T-independent form of PC resides solely within the B-1 B cell population.

There was no significant change in the levels of PC-specific Igh6b or $T15^+$ anti-PC in mice suppressed for the Igh6a allotype. However, in mice suppressed for Igh6b, the total Igh6a anti-PC response was essentially similar to control values, but it did not express the dominant T15 Id.

In an earlier study by Sigal and colleagues (1977), the splenic PC-specific precursor frequency of 4 to 5 day CB6 F1 neonates was significantly higher than that of BALB/c neonates of the same age. This indicated that PC-specific precursors arise earlier in CB6 F1 mice compared to those in parental BALB/c mice, although in this study, it was not determined whether these precursors expressed the Igh6b allotype. During the developmental window in which PC-specific precursors originate in CB6 F1 mice, it is likely that B cells expressing the Igh6b allotype are generated first, thereby inhibiting by feedback competition the selection of $T15^+$ PC-reactive B cells expressing the Igh6a allotype into the newly forming repertoire of F1 mice. In the mice suppressed for Igh6b, the failure of PC-specific Igh6a to express the dominant T15 Id may also be due to the inactivation by suppression of the earlier arising regulatory fetal-derived B cells that are normally predominantly Igh6b in F1 mice. In previous studies, we have shown that such B cells are involved in the development of T15 dominance (Vakil et al., 1986; Vakil and Kearney, 1988).

In each group of mice tested, the Ab response to the T-dependent form of PC was almost identical in all aspects to the Ab response to the T-independent form of this Ag. These results, as well as those of Taki and colleagues (1992), indicate that the ability to produce an Ab response to this Ag resides solely within the B-1 B cell subset regardless of how the Ag is presented to the B cell.

The responses of Igh6a-suppressed mice indicated that B-2 B cells were able to respond to DEX and that the response was of the dominant J558 Id. These results contrast with previous studies that suggested that DEX-specific responses arose from B-1 B cells (Förster and Rajewsky, 1987). However, we cannot exclude the possibility that there may have been other DEX-responsive Igh6a B cells in anatomically distinct sites.

Experiments performed by Förster and Rajewsky (1987) involved the transfer of BALB/c Igh6a adult bone marrow or peritoneal cells into CB.20 Igh6b newborn mice. When recipient mice were immunized with DEX, those that received adult peritoneal cavity B-1 B cells were able to respond to this Ag, although the idiotypic profile was not determined. Mice that received bone marrow as a source of progenitors for B-2 B cells were unable to respond to DEX. Additionally, other previous studies (Weiler et al., 1985) demonstrated active suppression of the DEX-specific Igh6a Ab response by Igh6b lymphocytes when BALB/c bone marrow was transferred into nude mice expressing the Igh6b allotype on a BALB/c genetic background. It is likely that the demonstrated suppressive action of host Igh6b lymphocytes would explain the lack of donor Igh6a DEX-specific Ab response in the mice receiving bone marrow cells in the cell-transfer experiments (Förster and Rajewsky, 1987) similar to that observed by Weiler and colleagues (1985). However, because the peritoneal cavity is not seeded with lymphocytes until 8 days after birth (Hamilton and Kearney, in preparation), there are no Igh6b lymphocytes in the peritoneal cavity of recipient mice at the time of peritoneal cell transfer. Therefore, there are no Igh6b peritoneal lymphocytes to compete for the seeding of the DEX-specific Igh6a precursors contained within the transferred peritoneal B-1 cells. Thus, the combined results of these transfer experiments may be interpreted as another example of the clonal competition that occurs in the establishment of the peritoneal population of B-1 cells.

In other experiments, anti-IL-10 treatment completely ablated the response to DEX in BALB/c mice, suggesting that the B-2 B cells remaining in the B-1-depleted mice did not respond to DEX (Ishida et al., 1992). Coadministration of anti-IL-10 and anti-$\gamma$ interferon reduced the ability of anti-IL-10 to deplete mice of peritoneal B-1 cells. The mechanism responsible for the depletion of peritoneal B-1 B cells in these mice appears to involve multiple cell–cytokine interactions. Therefore, the inability of B-2 B cells to respond to DEX in mice treated with anti-IL-10 Ab may be due to complex cell–cytokine interactions as yet undefined.

In Igh6b-suppressed mice, the majority of the Igh6a Ab response to DEX expressed the normally minor M104E Id. It was of interest that the M104E Id values surpassed the total anti-DEX response. We have seen this occasionally in other experiments in BALB/c mice, and it may reflect idiotype expressed on other than DEX-specific antibody. Our previous ontogenic studies have demonstrated that the first detectable DEX-specific B cells express the M104E Id, whereas J558$^+$ DEX-specific B cells arose later in ontogeny and then dominated the response in adults (Stohrer and Kearney, 1984). Similar to the results observed with PC, it is likely that in normal F1 mice, proposed regulatory B cells expressing the Igh6b allotype that are generated first promote the expansion of J558$^+$ DEX-specific B cells expressing the Igh6a allotype at the expense of the M104E Id$^+$ B cells. However, in the F1 mice suppressed for Igh6b, the failure to generate DEX-specific, J558$^+$ Igh6a may be due to the failure of these B cells to be selected because the earlier arising Igh6b regulatory B cells are absent or functionally inactivated as a result of the allotype-specific suppresion. Therefore, the earliest generated M104E$^+$ DEX-specific B cell precursors would remain as the dominant clones.

The independent suppression of the maternal or paternal IgM allotype in F1 mice provided a means to investigate the contribution of B cell subsets to serum IgM levels and specific Ab responses. These results indicate a division of Ab responsiveness to certain Ag between B-1 and B-2 B cell subsets and that the depletion of B-1 B cells early in development appears to influence the idiotypic profile of the Ab response from B-2 B cells. Properly chosen parental strains could provide a usable model for the role of early interactions between these B cell subsets in other Ag systems.

### MATERIALS AND METHODS

### Animals

BALB/c ($IgC_H{}^a$) and C57BL/6 ($IgC_H{}^b$) mice were obtained from Jackson Laboratories (Bar Harbor, ME) and bred in our animal facilities to produce the BALB/c×C57BL/6 (CB6) and C57BL/6×BALB/c (B6C) F1 mice used herein. There were no differences in Ab responses from either CB6 or B6C F1 mice. Results observed only in CB6 F1 mice are indicated in the text. Adult F1 mice were analyzed by serologic tests for viral and mycoplasma infections, *in vitro* cultures for bacterial and mycoplasma pathogens, and histologic examination for evidence of disease, as previously described (Parker et al., 1989). All results were negative.

### Antibodies

The mouse anti-mouse Igh6a mAb-producing hybridoma (RS-3.1) (Schuppel et al., 1987) was obtained from Dr. T. Imanishi-Kari. The mouse anti-mouse Igh6b mAb (Nishikawa et al., 1986) producing hybridoma (MB86) was obtained from Dr. A. Radbruch. Purified Ab was obtained by passing hybridoma culture supernatant over a goat anti-mouse $IgG_1$ (Southern Biotechnology Associates, Birmingham, AL) coupled CNBr-activated sepharose 4B (Pharmacia, Piscataway, NJ) affinity column, followed by elution with 0.1 M glycine/HCl (pH 2.8). The purified Ab was then dialyzed against PBS and filter sterilized for injection. Biotinylation of Ab was performed using standard protocols (Pierce, Rockford, IL). Conjugation of RS-3.1 and MB86 to FITC was carried out by Southern Biotechnology Associates. FITC-conjugated AF6-78.25 (Stall and Loken, 1984), unlabeled R6-71, and biotinylated R40-97 were purchased from Pharmingen (San Diego). FITC-conjugated goat anti-mouse IgM, streptavidin-phycoerythrin (SA-PE), and streptavidin-Texas Red (SA-TR) were purchased from Southern Biotechnology Associates. All other mAb used in this study are described in Table 2.

### Suppression Regimen

Mating of BALB/c and C57BL/6 mice produced the $IgC_H{}^{a/b}$ allotype heterozygous F1 mice used in this study. The suppression regimen has been previously described (Lalor et al., 1989b). Briefly, neonatal mice were given i.p. injections of 50 μg of either RS-3.1 or MB86 in PBS within 24 hr of birth. A second injection of 100 μg of the appropriate Ab was given i.p. at 7 days after birth followed by a final injection of 150 μg at 14 days after birth. The Ab, when administered at this dosage (300 μg total), is detectable in the circulation for at least 5 to 6 weeks (Lalor et al., 1989b). Control F1 mice received PBS alone.

### ELISA Analysis of Circulating Levels of Igh6 Allotype-Specific Ab

Mice were bled at 4, 5, and 6 weeks after birth and serum levels of RS-3.1 and MB86 determined using

TABLE 2
mAb Used in this Study

| Antibody Name | Specificity | Isotype | Source |
|---|---|---|---|
| RS-3.1 | Igh6a | mouse $\gamma_1$k | T. Imanishi-Kari; Schuppel et al., 1987 |
| MB86 | Igh6b | mouse $\gamma_1$k | A. Radbruch; Nishikawa et al., 1986 |
| AF6-78.25 | Igh6b | mouse $\gamma_1$k | Stall and Loken, 1984 |
| 14.8 | B220 | rat $\gamma_{2b}$k | Kincade et al., 1981 |
| 11-26c | mouse IgD | rat | D. Bole |
| M1/70.15.11.5 | Mac-1 (CD11b) | rat $\gamma_{2n}$k | Springer et al., 1979 |
| 53.7.313 | mouse CD5 | rat $\gamma_{2a}$k | Ledbetter and Herzenberg, 1979 |
| R52.120 | mouse IL5R | rat $\gamma_1$k | R. Palacios; Rolink et al., 1989 |
| B3B4 | mouse FcεR | rat $\gamma_{2a}$k | M. Kehry |
| R6-71 | mouse IgM | rat $\gamma_1$k | Pharmingen |
| R40-97 | mouse IgM | rat $\gamma_{2c}$k | Pharmingen |
| BH8 | PC | mouse μk | Pollok et al., 1984 |
| AB1-2 | T15 Id | mouse $\gamma_1$k | Kearney et al., 1983 |
| TC139-2 | T15 Id | rat | J. Kenny; Desaymard et al., 1984 |
| H1-3 | α1-3 DEX | mouse μk | M. Elliott |
| EB3-7 | J558 Id | mouse $\gamma_1$k | Stohrer et al., 1983 |
| M104E | α1-3 DEX | mouse $\mu\lambda_1$ | J. Eldridge |
| SJL18.1 | M104E | mouse μk | Stohrer et al., 1983 |

a quantitative ELISA. Ninety-six-well microtiter plates (Costar, Cambridge, MA) were coated with 2 μg/ml of a monoclonal IgM Ab of either the Igh6a or Igh6b allotype for 4 hr at 37°C. PBS containing 1% BSA and 0.1% sodium azide was then used as a blocking agent. After washing with PBS, the plates were incubated at 4°C overnight with serial dilutions of serum samples from individual mice or known amounts of RS-3.1 or MB86, which served as standards for the detection of each in the serum of suppressed mice. The plates were then washed with PBS, followed by incubation with alkaline phosphatase-labeled goat anti-mouse $IgG_1$ Ab (Southern Biotechnology Associates) at 37°C for 2hr. Color was developed using p-nitrophenyl phosphate (Sigma, St. Louis) as a substrate. The $OD_{405}$ absorbance values were read using a Titertek Multiscan PLUS (FLOW Laboratories, McClean, VA). Serum concentrations of RS-3.1 and MB86 were calculated using the ELISA LITE computer program (Meddata, New York).

### Immunofluorescence Staining of Peripheral Lymphocytes

Following isolation of single-cell suspensions of CB6 F1 spleen or peritoneal cavity, 2 × $10^5$ cells were incubated with either FITC-conjugated goat anti-mouse IgM, RS-3. 1-FITC, or AF6-78.25-FITC for 20 min. The cells were washed in PBS containing 1% crystallized BSA (Sigma) and 0.05% sodium azide, and then incubated with either 14.8-biotin, 11-26c-biotin, M1/70.15.11.5-biotin, R52.120-biotin, B3B4-biotin, or 53.7.313-biotin followed by SA-PE. The cells were then washed and analyzed by flow cytometry using the FACScan Consort 30 program (Becton Dickinson, Mountain View, CA).

### Immunofluorescence Staining of Fixed Tissues

Mice were killed by cervical dislocation and their spleens removed. Approximately one-third of each spleen was immersed in O.C.T. embedding compound (Miles Scientific, Elkhart, IN) and snap frozen in a plastic 'boat' floated on the surface of liquid nitrogen. Where possible, sections were cut immediately; otherwise, tissue blocks were stored at −70°C. Four micron sections were cut using a Minotome cryostat (IEC, Needham Heights, MA).

Following fixation, cryostat sections were allowed to air dry. After overnight incubation in a desiccator at 4°C, the tissue section was covered with 100 μl MB86-FITC plus RS-3.1-biotin, and the slide was placed in a humidified chamber for 20 min at room temperature. The section was then washed with PBS and subsequently incubated with 100 μl SA-TR for 20 min as before. The slides were then washed a final time and cover slips were mounted using Elvanol (Southern Biotechnology Associates). Sections were examined using a fluorescence microscope (Leitz, Germany).

### ELISA Analysis of Serum IgM

Mice were bled at 4, 5, and 6 weeks after birth and at various other times as mentioned in the text. The serum levels of Igh6a and Igh6b were determined using a quantitative ELISA, as described before with the following modifications. Plates were coated with 2 μg/ml of either RS-3.1 or MB86 for 4 hr at 37°C, thus permitting the detection of serum Igh6a and Igh6b levels, respectively. Known quantities of mAb that express either the Igh6a or Igh6b allotypic determinant were used as standards.

### Immunization Protocol

A heat-killed preparation of *S. pneumoniae* (strain R36A) was prepared as follows. R36A was grown in 3% Todd-Hewitt broth containing 0.5% yeast extract at 37°C until an $OD_{500}$ of 0.6 was reached. The bacteria were then heat killed at 56–60°C for 1 hr followed by extensive washing with cold PBS. An $OD_{420}$ was taken to quantitate the number of heat-killed bacteria per milliliter of solution. Dextran B-1355S (35% $\alpha$1–3 linkages), derived from *L. mesenteroides*, was a gift from Dr. M. Slodki. Approximately 6 to 14 weeks after birth, allotype-suppressed mice were given i.p. injections of 2 × $10^8$ R36A and 100 μg Dextran B-1355S in saline and bled 7 days later. Following a 3 to 4 week rest period, the same mice were given s.c. injections of 50 μg keyhole limpet hemocyanin (KLH) (Calbiochem-Behring Corp, LaJolla, CA) dissolved in sterile PBS. After 14 days, the mice were then administered i.p. injections of 100 μg PC-KLH (provided by Dr. J. Eldridge) in PBS and bled 7 days later.

### Antigens Used for ELISA

PC- and DEX-BSA were prepared as described elsewhere (Eisen, 1964; Chesebro and Metzger, 1972).

### ELISA Analysis of Serum Ab Responses

PC- and DEX-specific Ab in sera of immunized mice were determined using a quantitative ELISA as described before with the following variations. Plates were coated with 2 μg/ml of either PC-BSA, DEX-BSA, or monoclonal anti-Id Ab (listed in Table 2) for 4 hr at 37°C. Following incubation with serum samples or mAb standards, plates were incubated with either alkaline phosphatase-labeled goat anti-mouse IgM for 2 hr at 37°C or RS-3.1-biotin, MB86-biotin, or R40-97-biotin for 4 hr at 37°C. Subsequent to washing with PBS, plates that received biotin-labeled Ab were incubated with streptavidin-alkaline phosphatase (Southern Biotechnology Associates) overnight at 4°C. The concentration of Ab was calculated using ELISA LITE.

### ACKNOWLEDGMENTS

This work has been supported in part by NIH grants Al 18742, Al 30879, and CA 13148. The authors would like to thank Dr. N. Davidson for the preparation of tissue sections, Drs. N. Davidson and P. Burrows for critical evaluation of the manuscript, and A. Brookshire for preparation of the manuscript.

*(Received November 1, 1993)*

*(Accepted November 24, 1993)*

### REFERENCES

Blomberg B., Geckeler W. R., and Weigert M. (1972). Genetics of the antibody response to dextran in mice. Science **166:** 178–189.

Bos N. A., Meeuwsen C. G., Van Wijngaarden P., and Benner R. (1989). B cell repertoire in adult antigen-free and conventional neonatal BALB/c mice. II. Analysis of antigen-binding capacities in relation to $V_H$ gene usage. Eur. J. Immunol. 19: 1817–1822.

Cancro M. P., Sigal N. H., and Klinman N. R. (1978). Differential expression of an equivalent clonotype among BALB/c and C57BL/6 mice. J. Exp. Med. **147:** 1–12.

Chesebro B., and Metzger H. (1972). Affinity labeling of a phosphorylcholine binding mouse myeloma protein. Biochemistry **11:** 766–771.

Cooper M. D., Kearney J. F., Gathings W. E., and Lawton A. R. (1980). Effects of anti-Ig antibodies on the development and differentiation of B cells. Immunol. Rev. **52:** 29–53.

Cosenza H., and Köhler H. (1972). Specific inhibition of plaque formation to phosphorylcholine by antibody against antibody. Science **176:** 1027–1029.

Desaymard C., Guisti A. M., and Scharff M. D. (1984). Rat anti-T15 monoclonal antibodies with specificity for $V_H$ and $V_H$–$V_L$ epitopes. Mol. Immunol. **21:** 961–967.

Eisen H. N. (1964). Preparation of purified anti-2,4-dinitrophenyl antibodies. Methods Med. Res. **10:** 94–100.

Eskinazi D. P., Knight K. L., and Dray S. (1979). Kinetics of escape from suppression of Ig heavy chain allotypes in multiheterozygous rabbits. Eur. J. Immunol. **9:** 276–283.

Förster I., and Rajewsky K. (1987). Expansion and functional activity of Ly-1$^+$ B cells upon transfer of peritoneal cells into allotype-congenic, newborn mice. Eur. J. Immunol. **17:** 521–528.

Hardy, R. R. (1992). Variable gene usage physiology and development of Lyl$^+$ (CD5$^+$) B cells. Cur. Opin. Immunol. **4:** 181–185.

Harrison M. R., and Mage R. G. (1973). Allotype suppression in the rabbit. I. The ontogeny of cells bearing immunoglobulin of paternal allotype and the fate of these cells after treatment with antiallotype antisera. J. Exp. Med. **138:** 764–774.

Herzenberg L. A., Stall A. M., Lalor P. A., Sidman C., Moore W. A., Parks D. R., and Herzenberg L. A. (1986). The Lyl B cell lineage. Immunol. Rev. **93:** 81–102.

Ishida H., Hastings R., Kearney J., and Howard M. (1992). Continuous anti-interleukin 10 antibody administration depletes mice of Ly-1 B cells but not conventional B cells. J. Exp. Med. **175:** 1213–1220.

Kearney J. F., Pollok B. A., and Stohrer R. (1983). Analysis of idiotypic heterogeneity in the anti-α1-3 dextran and anti-phosphorylcholine responses using monoclonal anti-idiotype antibodies. Ann. N. Y. Acad. Sci. **418:** 151–170.

Kincade P. W., Lee G., Watanabe T., Sun L., and Scheid M. P. (1981). Antigens displayed on murine B lymphocyte precursors. J. Immunol. **127:** 2262–2268.

Lalor P. A., Herzenberg L. A., Adams S., and Stall A. M. (1989a). Feedback regulation of murine Ly-1 B cell development Eur. J. Immunol. **19:** 507–513.

Lalor P. A., Stall A. M., Adams S., and Herzenberg L. A. (1989b). Permanent alteration of the murine Ly-1 B repertoire due to selective depletion of Ly-1 B cells in neonatal animals. Eur. J. Immunol. **19:** 501–506.

Ledbetter J. A., and Herzenberg L. A. (1979). Xenogeneic monoclonal antibodies to mouse lymphoid differentiation antigens. Immunol. Rev. **47:** 63–90.

Lieberman R., Potter M., Mushinski E. B., Humphrey W. Jr., and Rudikoff S. (1974). Genetics of a new $IgV_H$ (T15 idiotype) marker in the mouse regulating natural antibody to phosphorylcholine. J. Exp. Med. **139:** 983–1001.

Mage R. G. (1967). Quantitative studies on the regulation of expression of genes for immunoglobulin allotypes in heterozygous rabbits. Cold Spring Harbor Symp. Quant. Biol. **32:** 203–210.

Masmoudi H., Mota-Santos R., Huetz F., Coutinho A., and Cazenave, P.-A. (1990). All T15 Id-positive antibodies (but not the majority of $V_HT15^+$ antibodies) are produced by peritoneal CD5$^+$ B lymphocytes. Int. Immunol. **2:** 515–520.

Nishikawa S., Sasaki Y., Kina T., Amagai T., and Katsura Y. (1986). A monoclonal antibody against Igh6-4 determinant. Immunogenetics **23:** 137–139.

Parker R. F., Davis J. F., and Cassell G. H. (1989). Short-term exposure to nitrogen dioxide enhances susceptibility to murine respiratory mycoplasmosis and decreases intrapulmonary killing of *Mycoplasma pulmonis*. Amer. Rev. Respir. Dis. **140:** 502–512.

Pollok B. A., Vakil M., Kearney J. F., and Perry R. P. (1984). A biological consequence of variation in the site of D-$J_H$ gene rearrangement. Nature **311:** 376–379.

Raman C. and Knight K. L (1992). CD5$^+$ B cells predominate in peripheral tissues of rabbit. J. Immunol. **149:** 3858–3864.

Rolink A., Melchers F., and Palacios R. (1989). Monoclonal antibodies reactive with mouse IL-5 receptor. J. Exp. Med. **169:** 1693–1701.

Schuppel R., Wilke J., and Weiler E. (1987). Monoclonal anti-allotype antibody towards BALB/c IgM. Analysis of specificity and site of a V-C crossover in recombinant strain BALB-Igh-$V^a$/Igh-$C^b$. Eur. J. Immunol. **17:** 739–741.

Sher A., And Cohn M. (1972). Inheritance of an idiotype associated with the immune response of inbred mice to phosphorylcholine Eur. J. Immunol. **2:** 319–326.

Sigal N. H., Pickard A. R., Metcalf E. S., Gearhart P. J., and Klinman N. R. (1977). Expression of phosphorylcholine-specific B cells during murine development. J. Exp. Med. **146:** 933–948.

Springer T. A., Galfre G., Secher D. S., and Milstein C. (1979). Mac-1: A macrophage differentiation antigen identified by monoclonal antibody. Eur. J. Immunol. **9:** 301–306.

Stall A. M., and Loken M. R. (1984). Allotypic specificities of murine IgD and IgM recognized by monoclonal antibodies. J. Immunol. **132:** 787–795.

Stall A. M., Quintáns J., and Loken M. R. (1986). T15 idiotype expression in the murine response to phosphorylcholine is actively regulated by genes linked to the Igh-C locus. J. Immunol. **136:** 2689–2696.

Stohrer R., Lee M. D., and Kearney J. F. (1983). Analysis of the anti-$\alpha 1 \rightarrow 3$ dextran response with monoclonal anti-idiotypic antibodies. J. Immunol. **131:** 1375–1379.

Stohrer R., and Kearney J. F. (1984). Ontogeny of B cell precursors responding to $\alpha$1–3 dextran in BALB/c mice. J. Immunol. **133:** 2323–2326.

Taki S., Schmitt M., Tarlinton D., Förster I., and Rajewsky K. (1992). T cell-dependent antibody production by Ly-1 B cells. Ann. N. Y. Acad. Sci. **651:** 328–335.

Thomas-Vaslin V., Coutinho A., and Huetz F. (1992). Origin of $CD5^+$ B cells and natural IgM-secreting cells: Reconstitution potential of adult bone marrow, spleen and peritoneal cells. Eur. J. Immunol. **22:** 1243–1251.

Vakil M., and Kearney J. F. (1988). Regulatory influences of neonatal multispecific antibodies on the developing B cell repertoire. Int. Rev. Immunol. **3:** 117–131.

Vakil M., Sauter H., Paige C., and Kearney J. F. (1986). *In vivo* suppression of perinatal multispecific B cells results in a distortion of the adult B cell repertoire. Eur. J. Immunol. **16:** 1159–1165.

Weiler I. J., Sprenger R., and Weiler E. (1985). Nude mice are nonpermissive towards the anti-dextran response of congenic cells. Eur. J. Immunol. **15:** 1102–1106.

